# On Thermal and Electrodynamic Aspects of the Superconductive Transition Process

**DOI:** 10.3390/ma17010254

**Published:** 2024-01-03

**Authors:** J. E. Hirsch

**Affiliations:** Department of Physics, University of California, San Diego, La Jolla, CA 92093-0319, USA; jhirsch@ucsd.edu

**Keywords:** Meissner effect, Alfven’s theorem, Faraday’s law, Lorentz force, holes

## Abstract

In a classic paper of 1960, W. H. Cherry and J. I. Gittleman discussed various thermal and electrodynamic aspects of the superconductive transition process relevant to practical applications. In a section of the paper that has remained unnoticed, they proposed a physical model for the Meissner effect. Earlier in 1940–1943, in work that has also remained unnoticed, K. M. Koch had introduced related physical ideas to explain the Meissner effect. Still earlier in 1937, J. C. Slater proposed a model to explain the perfect diamagnetism of superconductors. None of these ideas are part of the conventional London-BCS understanding of superconductivity, yet I will argue that they are essential to understand the Meissner effect, the most fundamental property of superconductors. The unconventional theory of hole superconductivity unifies and extends these ideas. A key missing element in the conventional theory as well as in these early theories is electron-hole asymmetry. A proper understanding of the Meissner effect may help with practical applications of superconductors, as well as to find new superconducting materials with desirable properties.

## 1. Introduction

The Meissner effect is the *process* by which a normal metal develops a supercurrent and expels an interior magnetic field as it transits from the normal into the superconducting state. Any theory that claims to describe real superconducting materials has to have the physical elements that are necessary to describe this process, as well as the reverse process, the transition of a superconductor carrying a Meissner current into the normal state with no current.

Key questions about the Meissner effect that have to be answered are: (i) What is the force that causes the development of the supercurrent that is generated to expel and ultimately exclude the magnetic field? (ii) How does the growing supercurrent overcome the counter-emf resulting from Faraday’s law that tries to slow it down? (iii) How is the momentum of the supercurrent compensated so that momentum conservation is not violated? Similarly, key questions about the reverse process, the superconductor to normal transition in a magnetic field, are: (i) How does the supercurrent stop before the material develops resistance [1], which is necessary to ensure that the transition is reversible? (ii) How is the momentum of the supercurrent transfered to the body as a whole without generation of Joule heat and associated entropy production? (iii) How does the stopping supercurrent overcome the Faraday counter-emf that propels it to continue flowing as the magnetic flux penetrates?

These basic and fundamental questions have been by and large ignored in the superconductivity literature, which is extremely surprising. Over the past 20 years, we have provided answers to these questions [2,3] based on the unconventional theory of hole superconductivity [4], proposed to apply to all superconducting materials [5]. In short and qualitatively, the force that drives the Meissner current is the magnetic Lorentz force acting on electrons moving outward towards the surface during the transition. Backflowing electrons of negative effective mass transfer their azimuthal momentum to the body as a whole without scattering processes, thus ensuring that the process is thermodynamically reversible. Locally, outward motion of electrons results from enlargement of electronic orbits. The same physics acting in reverse explains the superconductor to normal transition in a field.

The conventional (BCS) theory of superconductivity [6] does not have the physical elements necessary to describe these processes. It is indifferent to whether the charge carriers have positive or negative effective mass, or negative or positive charge, and it does not describe radial charge flow in the transition process nor large orbits in the superconducting state. For this reason, we have argued that the conventional theory cannot describe real superconductors, since all superconductors exhibit a Meissner effect. Nevertheless, the theory continues to be used to describe existing and predicted new superconductors [7].

There are three works in the early literature that addressed some of these questions and provided partial answers to them, by J. C. Slater [8], K. M. Koch [9,10,11] and W. H. Cherry [12]. They were independent of each other. None of them is cited in the classic BCS paper [13] nor in any book on superconductivity (except Ref. [3]). We discuss them and their relation with the theory of hole superconductivity in what follows.

## 2. Cherry–Gittleman Paper

In 1960, W. H. Cherry and J. I. Gittleman addressed the problem of current quenching in superconductors [12], important for technological applications. They considered a superconducting wire and analyzed the various processes that occur when the system makes a transition to the normal state when the current exceeds the critical current. They pointed out that Joule heat generated by eddy currents in the portions of the material that initially become normal have an important effect on the transition process. Notably, they do not discuss the fact that the process of supercurrent stopping when a portion of the material becomes normal could also generate Joule heat if the supercurrent were to stop by onset of resistance as one might expect. The reason they did not presumably is that it had been shown conclusively by Keesom earlier [1,14] that the supercurrent stops before the system develops resistance, hence no Joule heat is generated in this process. This is also implicit in the BCS description of the thermodynamics of superconductors [6], but nobody has explained how it happens dynamically within that theory. We have argued that within the conventional theory the current can only stop through scattering processes that generate Joule heat [15], in contradiction with the thermodynamic description.

Sect. III of Ref. [12], titled “Electrodynamic aspects of the transition process”, deals with the Meissner effect. It was authored solely by W. H. Cherry (a footnote to Sect. III in Ref. [12] reads *“One of the authors of this paper (W. H. C.) is the sole author of this section, and wishes to acknowledge much help from consultations with J. I. Gittleman, G. A. Morton and R. H. Parmenter”*). It is noteworthy that none of the 70 papers that cited Ref. [12] make any reference to Sect. III. Cherry pointed out the following:

On the London equation: *“It does not account for the Meissner-Ochsenfeld effect, that is the sudden appearance of almost perfect diamagnetism upon the instant following the transition into the superconducting state. Instead it would predict the “freezing in” of magnetic flux”*. He concludes that the Meissner current is *“induced through the interaction of the magnetic field with some mechanism active in the material during the transition”*, *“What is important, however, is that the mechanism is active only during the transition, and although the supercurrents it generates will persist afterward, once the transition is over the mechanism itself is gone, and no new diamagnetic currents are produced by it, even though the magnetic field should change”*.

Later in the section, he states: *“as a matter of general observation, the transition into the superconducting state, at least in the presence of a magnetic field, takes place by a very pronounced nucleation and growth process, it is conceivable that this growth, involving as it does the motion of a boundary, provides just the mechanism sought”*. And he proposes the following mechanism: *“We suppose that the material, presumably in a magnetic field, as it approaches the conditions in which the superconducting fluid will form passes at first into the superconducting state only in the neighborhood of points where the conditions or characteristics of the material are slightly more favorable…These regions now act as sources of the superconducting fluid which flows or diffuses (not necessarily a random-walk process) outwards into regions which are slightly less favorable to the formation of the fluid…”*. It continues: *“Now in this process of outward diffusion, the fluid passes across the lines of magnetic induction and correspondingly receives lateral thrusts which bend the fluid into current paths surrounding the central source regions and the regions immediately adjacent. These currents, as is easily seen, are in such directions as to weaken the magnetic fields on the insides, and hence the inside regions not formerly able to generate the superconducting fluid can now do so, or if previously able slowly, can now do so more rapidly, thereby contributing to the further outward diffusion and the further expulsion of flux. To compensate for the slight charge imbalance resulting from this outward diffusion, an inward diffusion of the normally conducting fluid will take place. However, the latter, being closely coupled to the lattice, will perhaps move more slowly and in any event will transfer the thrusts it receives from the magnetic field to the lattice, so that there will be negligible counter diamagnetic currents generated. At the same time that the outward diffusion into the magnetic field creates peripheral diamagnetic currents, the interior acquires the properties of a bulk perfect conductor. The diamagnetic currents persisting there from the previous growth stage are neutralized by the counter electric field produced by the new currents”*.

This remarkably vivid description of the physics proposed to be involved in the superconducting transition process in a magnetic field is in complete agreement with what is predicted by the theory of hole superconductivity [2,3]. This physics is completely absent in the conventional BCS description. Important missing elements to these insightful remarks are what is the sign of the charge of the fluid diffusing outward and inward, and any suggestion on the mechanism by which the inwardly diffusing fluid would *“transfer the thrusts it receives from the magnetic field to the lattice”*.

## 3. Koch’s Papers

**Figure 1:**
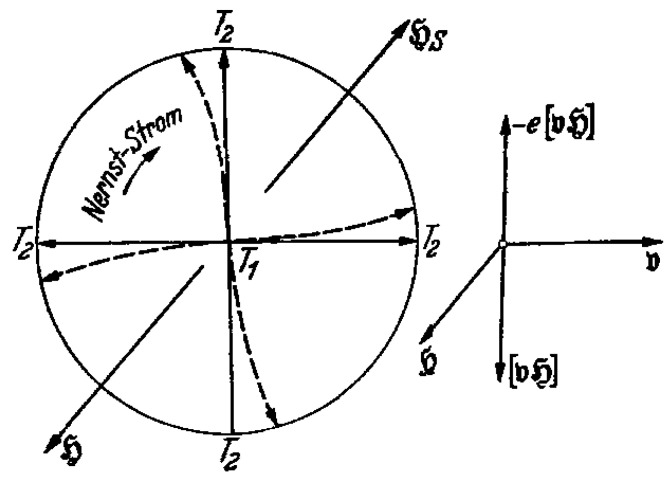
From Koch’s 1940 paper [9]. Its caption (translated from German) reads: *“Electrons moving from the interior outward due to the temperature gradient ($T_{1}>T_{2}$) are deflected by the magnetic field H. The figure on the side shows the direction of the Lorentz force for an electron moving to the right. The resulting circular current is depicted in the Figure in the conventional sense (positive charge carriers). Its magnetic field $H_{S}$ is in direction opposite to that of the primary field (H)”*. “Nerst-Strom” in the figure means “Nerst current”.

In 1940, K. M. Koch published a paper [9] titled *“Versuch einer elektronenphysikalischen Deutung des Meissner-Ochsenfeld-Effektes”*, meaning *“Attempt at an electrophysical interpretation of the Meissner-Ochsenfeld effect”*. In this paper, he emphasized the crucial fact that in the derivation of London’s equation, infinite conductivity is assumed from the outset, hence it cannot give any information on the transition process, hence on the mechanism of the Meissner effect. He then proposed that during the transition a thermal gradient would exist, with the interior of the body at higher temperature than the boundary, and that a resulting radial thermoelectric charge outflow would be deflected through the magnetic Lorentz force to give rise to a surface current screening the interior magnetic field. This is the first time in the scientific literature where outgoing charge flow was proposed as an explanation of the Meissner effect. Figure 1 from Koch’s paper shows the physics. Note that this physics would work equally well whether the outflowing charge is electrons or holes, i.e., whether the thermoelectric power of the material is negative or positive.

In a follow-up paper three years later [10], titled (translated from German) *“A new attempt at the interpretation of the Meissner effect and the “germ” theory of superconductivity”*, Koch abandons the thermoelectric interpretation and instead points out that the transition to the superconducting state is likely to occur through nucleation, growth and ultimate merging of initially small domains (“germs”). He points out that as a “germ” grows, so does the magnetic flux through it, and claims that this gives rise to an induced current that generates a magnetic field opposing the external field. However, in this paper he does not explicitly talk about outward charge flow being associated with the growth of domains, and in the absence of charge flow in fact no current would be induced.

Finally, in a later review article coauthored with the experimentalist E. Justi [11] Koch writes (translated from German):

*“Assuming that the transition N→S is in some way associated with electronic motion from the interior of the body to its surface - and we will immediately see that for the realization of this several different possibilities exist - one can see that that way a shielding current can be generated in the presence of a constant magnetic field. Considering for simplicity a body of cylindrical shape, with H parallel to the axis, we can see in Figure 53 that electrons are deflected from their radial motion by the magnetic field, so that they yield a circular current of orientation such that the primary field is weakened. One can easily compute that the such developed current must become a surface current through electrodynamic reasons and that (under the assumption of infinite conductivity) the weakening of the primary magnetic field would result in a complete cancellation of it, if the radial electron current persists for sufficiently long”.*

He continues to discuss his original thermoelectric explanation [9] for the outward charge flow and dismisses it for a variety of reasons, then writes [11], in reference to his second proposal [10]:

*“…we assume that the superconducting state develops from germs, that gradually grow and finally somehow merge. Thinking about the growth of these microdomains taking place in a constant magnetic field, one can see that the magnetic flux ϕ through the cross-section of these domains grows and this gives rise to an induction current that - according to elementary induction laws - cancel the primary flux. One can also think about this growth process as only the outer covering of these domains being occupied by superconducting electrons, and that this covering as it is joined by further accumulation of normal particles preserves its electrical connection. Then it is also essentially again so, that electrons - this time through a structural process - move centrifugally and are thus deflected by the magnetic field”*.

In other words, here Koch realized that his earlier proposal of domain growth without charge flow [10] did not give a mechanism for current development, so he amended it to include the necessary outflow of charge.

Thus, the physical explanations for the Meissner effect arrived at by K. M. Koch in the 1940’s and W. H. Cherry some 20 years later, are essentially identical: outward motion of electrons associated with the growth of superconducting domains in a normal matrix in the presence of a magnetic field gives rise, through the action of the magnetic Lorentz force on the outflowing electrons, to a surface current that cancels the interior magnetic field. Cherry’s explanation has the additional feature of describing backflow of charge, which is necessary since otherwise a huge charge imbalance would develop.

It is essentially certain that Cherry was unaware of Koch’s work on the subject: Koch’s paper on “germs” [10] has no citations, and the other Koch papers on the subject [9,11] have very few citations. I was unaware of the work of both of these authors when I developed an explanation of the Meissner effect that contains this physics based on the theory of hole superconductivity during the last 20 years.

Before delving into this, however, we should discuss another important precedent: Slater’s explanation for the perfect diamagnetism of superconductors [8].

## 4. Slater’s Paper

In 1937, J. C. Slater proposed [8] that the perfect diamagnetism of superconductors can be understood if electrons reside in *“orbits of order of magnitude of 137 atomic diameters”*. One hundred thirty-seven is the inverse of the fine structure constant

(1) $$\alpha =\frac{e^{2}}{\hbar c}∼\frac{1}{137}$$

The rationale for Slater’s proposal is as follows. The Larmor diamagnetic susceptibility for a solid with atomic density *n* and one electron per atom and electronic orbits perpendicular to the applied magnetic field is

(2) $$\chi =-\frac{ne^{2}}{4m_{e}c^{2}}<r^{2}>$$

in Gaussian units, where *e* and $m_{e}$ are the electron’s charge and mass, and $<r^{2}>$ is the average of the square of the radius of the electronic orbit. Perfect diamagnetism as occurs in a superconductor requires χ=−1/(4π), which implies

(3) $$<r^{2}>=\frac{m_{e}c^{2}}{\pi e^{2}n}$$

Assuming n=1/v, with $v=\left(4/3\right)\pi a_{0}^{3}$ the atomic volume for an atom of radius $a_{0}=\hbar^{2}/\left(m_{e}e^{2}\right)$, the Bohr radius, yields for the radius of the electronic orbits $r_{o}$

(4) $$r_{o}∼\sqrt{<r^{2}>}=1.15\times \left(137a_{0}\right)$$

justifying Slater’s proposal. The conventional theory of superconductivity does not describe such orbits. As we will discuss, they are essential to understand the Meissner effect within the theory of hole superconductivity. We were not aware of Slater’s paper when we came to that conclusion.

## 5. Meissner Effect in the Theory of Hole Superconductivity

The theory of hole superconductivity was developed over the past 35 years [4], starting in 1988 [16]. It was only beginning in the year 2003 that its implications for the understanding of the Meissner effect started to become apparent.

A fundamental aspect of the theory is electron-hole asymmetry, and in particular the fact that superconductivity originates in electronic energy bands that are almost full. Within this theory hole carriers are necessary for superconductivity to exist. This was clear from the outset [16]. In early work, we found that charge asymmetry would lead to an effective attractive interaction between holes resulting from their Coulomb interaction and the nature of the electronic wavefunctions in the solid state environment [17,18] and to pairing and superconductivity when the band is almost full [19,20].

**Figure 2:**
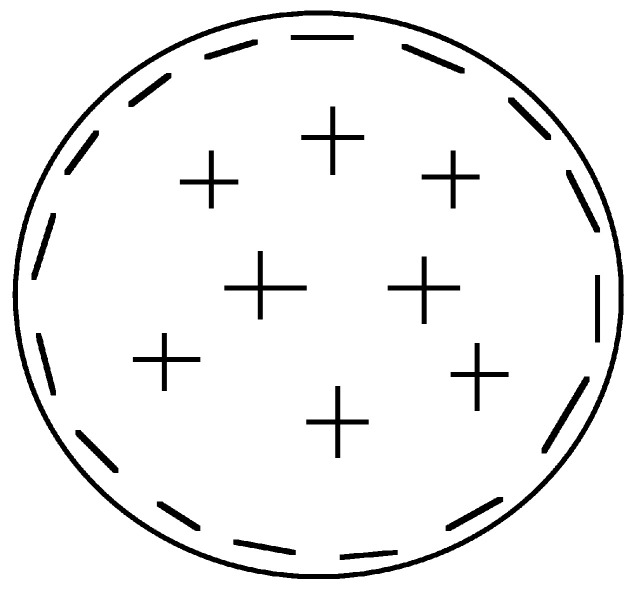
Schematic picture of the charge distribution in the ground state of a superconducting body, from Ref. [21] of 2001. The figure caption in Ref. [21] says *“Negative charge is expelled from the bulk to the surface”*, but no connection to the Meissner effect is made in that paper.

In the year 2001, I reached the conclusion that the fundamental charge asymmetry underlying the theory would cause superconductors to expel electrons from the interior towards the surface [21]. I predicted that the ground state of a superconductor would have a macroscopically inhomogeneous charge distribution, as shown in Figure 2, taken from my 2001 paper. At that time I was unaware of the fact that Koch had predicted charge expulsion as shown in Figure 1 (I discovered Koch’s papers in 2009 through a Google Scholar search).

**Figure 3:**
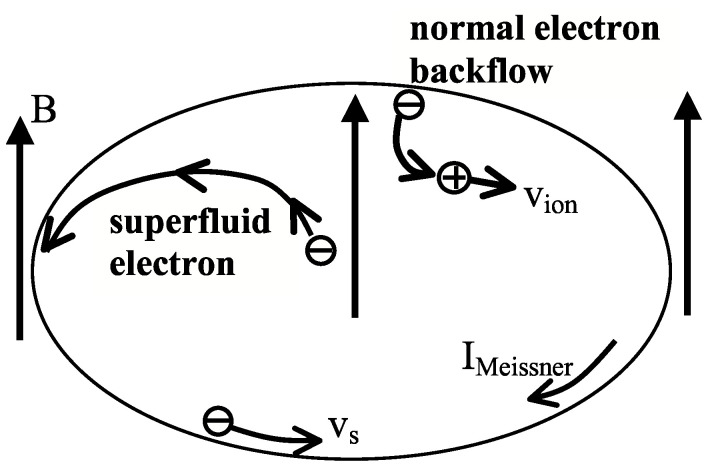
Schematic picture of the charge flow during the superconducting transition proposed to explain the Meissner effect, from Ref. [24] of 2008. The figure caption in Ref. [24] reads *“Superfluid electrons flow from the interior towards the surface and are deflected to the left by the magnetic field pointing up. Normal electrons backflow from the surface towards the interior and are deflected to the right by the magnetic field. The momentum in this normal current is transferred to the ions by collisions with impurities”*. It was only 8 years later that I realized that the last sentence in this caption is incorrect.

In 2003, for the first time, I made a connection between outflow of negative charge in the transition from the normal to the superconducting state and the Meissner effect [22,23] that unbeknownst to me had been proposed by Koch 60 years earlier. It was not until 5 years later that I came to the conclusion that outflow of negative charge would require the existence of backflow to restore approximate charge neutrality, as I discussed in a paper in 2008 [24]. Figure 3 shows the predicted behavior. At that time, I was unaware of the fact that Cherry had predicted that behavior in 1960 in his discussion of outward and inward diffusion of the conducting fluid during the transition process reviewed in Sect. 2 (I discovered the Cherry-Gittleman paper in 2017 through a Google Scholar search). Nor did I correctly understand at that time how the backflowing electrons transfer their momentum to the ions, as indicated in the caption of Figure 3.

The fact that expulsion of magnetic field requires radial charge flow can in fact be proven mathematically [25,26]; it is a consequence of Alfven’s theorem [27,28]. Consider the equation of motion for an electron in the superconducting fluid that follows from Newton’s and Maxwell’s equations

(5) $$\frac{d{\vec{v}}_{s}}{dt}=\frac{e}{m_{e}}\left(\vec{E}+\frac{{\vec{v}}_{s}}{c}\times \vec{B}\right)$$

where d/dt is the convective time derivative. It yields [29]

(6) $$\frac{\partial \vec{w}}{\partial t}=\vec{\nabla }\times \left({\vec{v}}_{s}\times \vec{w}\right)$$

where $\vec{w}$ is the generalized vorticity

(7) $$\vec{w}\left(\vec{r},t\right)=\vec{\nabla }\times {\vec{v}}_{s}\left(\vec{r},t\right)+\frac{e}{m_{e}c}\vec{B}\left(\vec{r},t\right).$$

The condition $\vec{w}\left(\vec{r},t\right)=0$ is equivalent to the London equation, describing the situation where the magnetic field is excluded from the interior. In the initial state with an applied magnetic field ${\vec{B}}_{0}$, $\vec{w}\left(\vec{r},t=0\right)=e/\left(m_{e}c\right){\vec{B}}_{0}$ is non-zero everywhere in the interior of the material. In cylindrical coordinates with $\vec{B}$ in the *z* direction $\vec{w}=w\hat{z}$ and Equation (6) is

(8) $$\frac{\partial w}{\partial t}=-\frac{1}{r}\frac{\partial }{\partial r}\left(rwv_{r}\right)$$

which shows that a *radial velocity* $v_{r}\neq 0$ of the fluid is a prerequisite for *w* to change in time. In the absence of a radial velocity, ∂w/∂t=0 and the magnetic field in the interior of the metal becoming superconducting would remain unchanged.

Also in 2008, I came to the conclusion that mesoscopic orbits of radius $2\lambda_{L}$, with $\lambda_{L}$ the London penetration depth, play a key role. I came to this conclusion through the following argument [30]: *“Assume the transition to superconductivity involves a radially outward motion of electrons to orbits of radius $r=2\lambda_{L}$. The electrons will acquire an azimuthal velocity $v_{\phi }$ as they move outward due to the magnetic Lorentz force $\vec{F}=\left(e/c\right)\vec{v}\times \vec{B}$”*. As shown in Ref. [30], the resulting azimuthal velocity for an orbit of radius *r* is

(9) $$v_{\phi }=-\frac{e}{2m_{e}c}\vec{r}\times \vec{B}$$

and for $r=2\lambda_{L}$

(10) $$v_{\phi }=-\frac{e\lambda_{L}}{m_{e}c}B\hat{\phi }$$

which is precisely the velocity of electrons in the Meissner current for a superconductor in an applied magnetic field *B* according to the conventional theory [6]. This is seen from the fact that the canonical momentum of an electron is

(11) $$\vec{p}=m_{e}\vec{v}-\frac{e}{c}\vec{A}.$$

In the superconducting state in a simply connected superconductor $\vec{p}=0$, and $A=\lambda_{L}B$ in a cylindrical geometry, from which Equation (10) follows.

**Figure 4:**
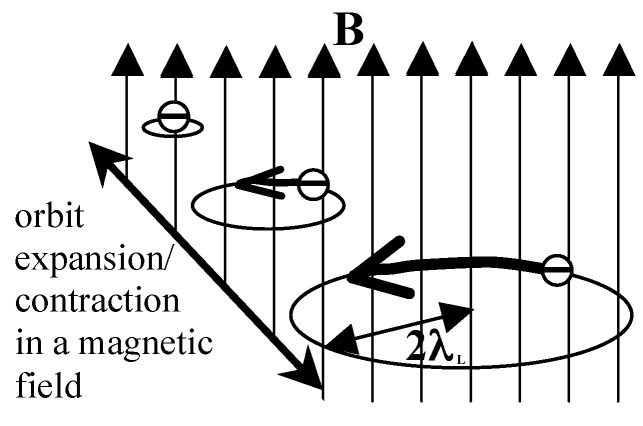
An electron expanding its orbit in the presence of a magnetic field B perpendicular to the orbit acquires an azimuthal velocity that generates a magnetic field opposite to the applied field.

This argument not only predicts that superconducting electrons reside in orbits of radius $2\lambda_{L}$ but also provides a dynamical explanation for how the electrons acquire the Meissner velocity Equation (10) in the process of going from normal to superconducting, as shown schematically in Figure 4. From Equation (2) for the diamagnetic susceptibility it is easy to see [24] that for $<r^{2}>={\left(2\lambda_{L}\right)}^{2}$ the susceptibility is −1/(4π), and for $<r^{2}>=k_{F}^{-2}$, with $k_{F}$ the Fermi wavevector, Equation (2) yields the Landau diamagnetic susceptibility of the normal metal. Thus, the *“some mechanism active in the material during the transition”* hypothesized by Cherry is precisely the expansion of the orbit from the microscopic radius $k_{F}^{-1}$ in the normal state to the mesoscopic radius $2\lambda_{L}$ in the superconducting state, which causes the electron to acquire an azimuthal velocity through the action of the Lorentz force. Conversely, shrinking of the orbit from the mesoscopic to the microscopic radius in the superconductor to normal transition provides, through the action of the Lorentz force, the mechanism sought by Keesom [1] for the supercurrent to stop without generation of Joule heat and entropy [31]. We should also point out that even in the absence of an applied magnetic field, electrons expanding their orbits acquire an azimuthal velocity through the spin-orbit interaction, in the opposite direction for the two members of a Cooper pair [30].

Note that $2\lambda_{L}$ orbits are closely related to Slater’s orbits hypothesized in 1937 [8] and discussed in Sect. 4. The London penetration depth follows from London’s equation as [6]

(12) $$\frac{1}{\lambda_{L}^{2}}=\frac{4\pi n_{s}e^{2}}{m_{e}c^{2}}$$

with $n_{s}$ the superfluid density. Hence,

(13) $${\left(2\lambda_{L}\right)}^{2}=\frac{m_{e}c^{2}}{\pi n_{s}e^{2}}$$

identical to Equation (3) if $n_{s}=n$ and $2\lambda_{L}=\sqrt{<r^{2}>}$. I was completely unaware of Slater’s paper [8] when I came to understand the relevance of $2\lambda_{L}$ orbits [24,30].

**Figure 5:**
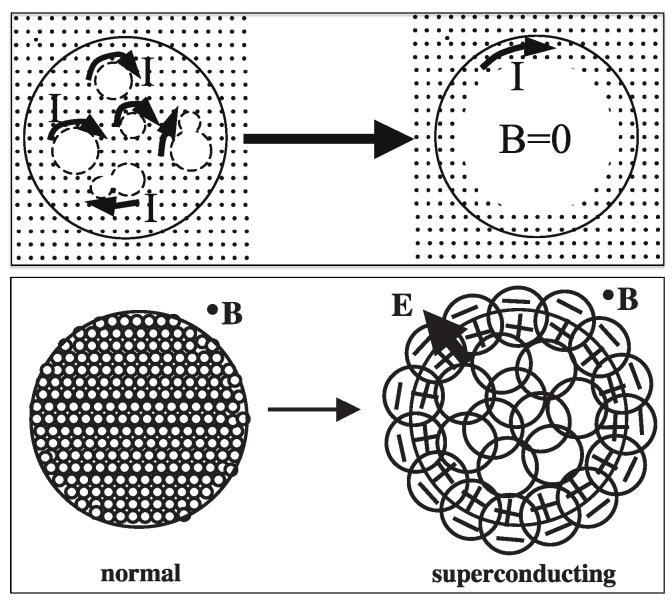
The top left panel, from Ref. [32], shows how superconducting domains with surface currents are created that exclude the magnetic field (black dots) from their interior; the top right panel shows the final state after the superconducting domains grow and merge. The bottom panel, from Ref. [24], shows a single domain where the enlarged orbits lead to expulsion of negative charge and a radial outgoing electric field.

**Figure 6:**
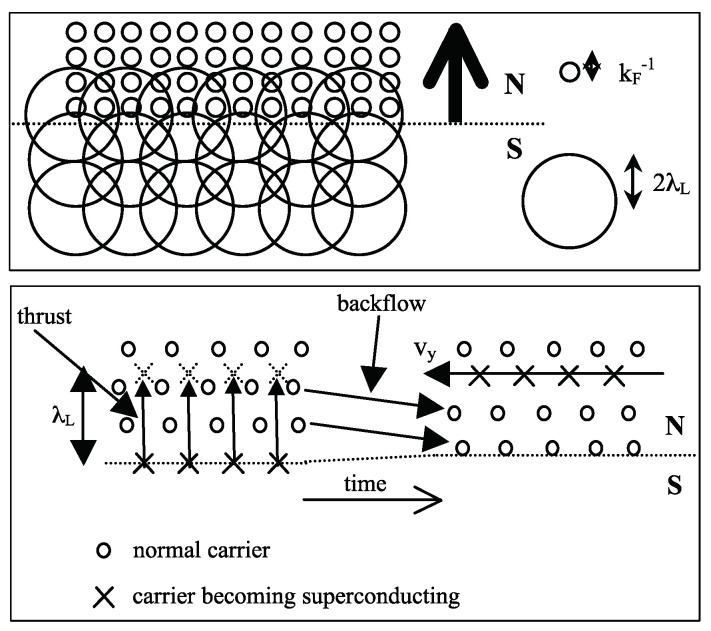
Upper panels show schematically orbits in the superconducting and normal regions. As normal electrons enter the superconducting region, their orbits expand causing a forward thrust of negative charge over a distance $\lambda_{L}$, as shown in the lower panel left side. This gives rise to a backflow of normal charge shown on the right side of the lower panel.

In Figure 5 top panel, from Ref. [32] of 2015, we show how the superconducting state evolves from growth and merging of domains. Figure 5 bottom panel, from Ref. [24], shows how expansion of the orbits leads to negative charge expulsion and a radial electric field at the boundary of the domain. In Figure 6, from Ref. [33] of 2016, we show how the phase boundary advances as the superconducting region grows, in a planar geometry for simplicity. Normal carriers expand their orbit as they become superconducting, and in the process extend their wavefunction into the normal region. This is equivalent to the charge thrusting forward a distance $\lambda_{L}$, as shown in the lower panel of Figure 6. This causes the backflow of normal carriers towards the superconducting region, as discussed by Cherry [12].

In the final superconducting ground state, electrons reside in overlapping orbits of radius $2\lambda_{L}$, with azimuthal velocity Equation (10) in the presence of a magnetic field B. The interior velocities cancel out, and only the velocities of electrons within a London penetration depth of the surface remain, giving rise to the Meissner current. That this is so is most simply seen from the following argument [24]: the angular momentum of the Meissner current flowing in a cylinder of radius R and height h within a London penetration depth of the surface is

(14) $$L=\left[m_{e}v_{\phi }R\right]\times n_{s}\times \left(2\pi R\lambda_{L}h\right)$$

where the factor in square parentheses is the angular momentum for one electron in the Meissner current (assuming $R>>\lambda_{L}$), and the factor in round parentheses is the volume where the Meissner current flows, i.e., the cylindrical shell of thickness $\lambda_{L}$ next to the surface. Equation (14) can also be written as

(15) $$L=\left[m_{e}v_{\phi }\left(2\lambda_{L}\right)\right]\times n_{s}\times \left(\pi R^{2}h\right)$$

describing the motion of all the electrons in orbits of radius $2\lambda_{L}$ in the entire volume $\pi R^{2}h$. The factor in square brackets is the angular momentum of one electron in its $2\lambda_{L}$ orbit.

**Figure 7:**
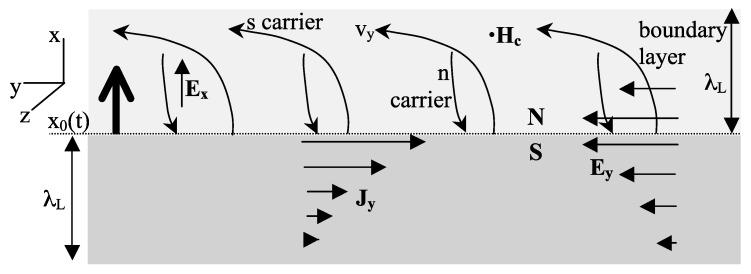
Motion of electrons becoming superconducting (s carriers) and backflowing normal electrons (n carriers) as the S-N phase boundary moves up in the figure. The magnetic Lorentz force acts to the left on s carriers and to the right on n carriers. However, the backflowing electrons are not deflected to the right as the figure shows. The explanation is given in Figure 8.

**Figure 8:**
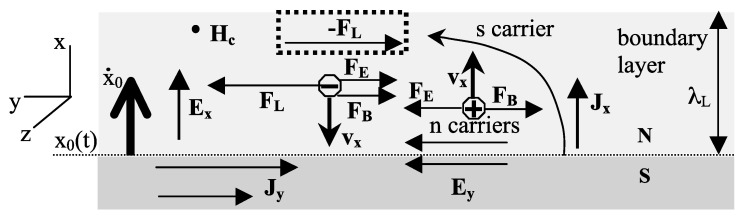
Explanation of how momentum is conserved in the normal to superconductor transition. Backflowing electrons of negative effective mass experience a force $F_{L}$ from the lattice to balance electric (from Faraday’s law) and magnetic forces acting on them. The resulting force $-F_{L}$ from electrons acting on the ions transfers momentum to the body as a whole.

**Figure 9:**
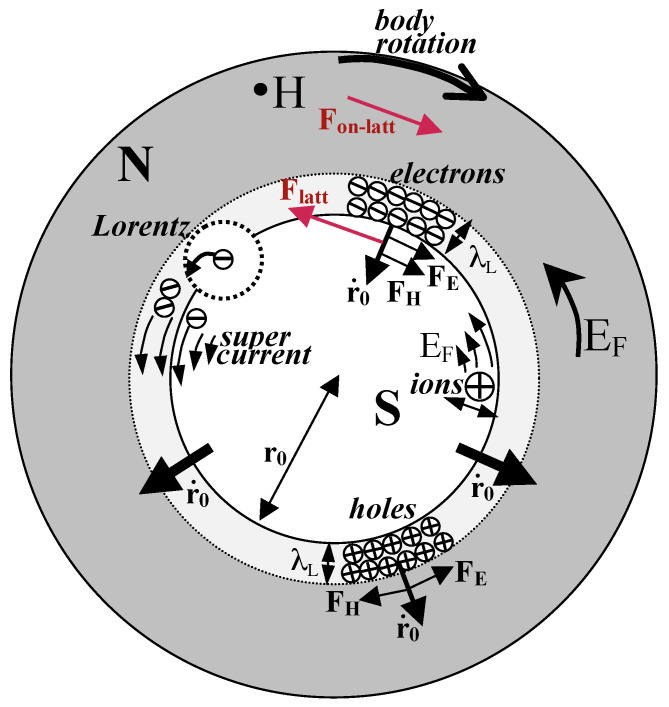
Meissner transition for a cylinder. The magnetic field points out of the paper, Meissner current flows clockwise. $E_{F}$ is the Faraday electric field. The backflowing carriers are shown both as electrons moving in (upper part) or equivalently as holes moving out (lower part). For holes, electric and magnetic forces $F_{E}$ and $F_{H}$ are balanced. For backflowing electrons, they are balanced by the force $F_{latt}$ exerted by the lattice on electrons with negative effective mass. Associated with it there is a force on the lattice, $F_{on-latt}$, that transfers momentum to the body that rotates with angular momentum equal and opposite to that of the Meissner current [34].

Figure 7 shows in more detail the motion of carriers. Electrons becoming superconducting thrust into the normal region and are deflected to the left by the Lorentz force (“s carrier”). It would appear that backflowing electrons (“n carrier”) would be deflected to the right by the Lorentz force, as shown in Figure 7. However that is not what happens. Because the Fermi level is close to the top of the band, normal carriers have hole-like character. If we think about backflowing electrons as forward-flowing holes, as shown in Figure 8, it is clear that the motion is perpendicular to the phase boundary, because electric and magnetic forces are exactly balanced. Figure 8 also shows how this can be understood in terms of backflowing electrons. The magnetic Lorentz force $F_{B}$ and the electric force due to the Faraday field both act in the same direction and are counterbalanced by a force that the lattice exerts on them, $F_{L}$. In turn, the electrons exert a force $-F_{L}$ on the lattice that transfers the same momentum to the lattice as the supercurrent acquires in the opposite direction. This is explained in quantitative detail in Ref. [34]. The transfer of momentum from electrons to the body as a whole can be understood as the Ampere force that results when hole carriers are the normal current carriers [34]. In Figure 9, we show the process in a cylindrical geometry with the applied magnetic field *H* coming out of the paper. The backflow is in fact not quite complete, and a small charge imbalance remains in the final state, as shown in Figure 2; the compensating azimuthal momentum for the excess negative charge near the surface is stored in the electromagnetic field [34]. The same processes with the arrows reversed occur in the transition from the superconducting to the normal state in the presence of a magnetic field and, in particular, explain how the supercurrent stops without entropy generation [15].

**Figure 10:**
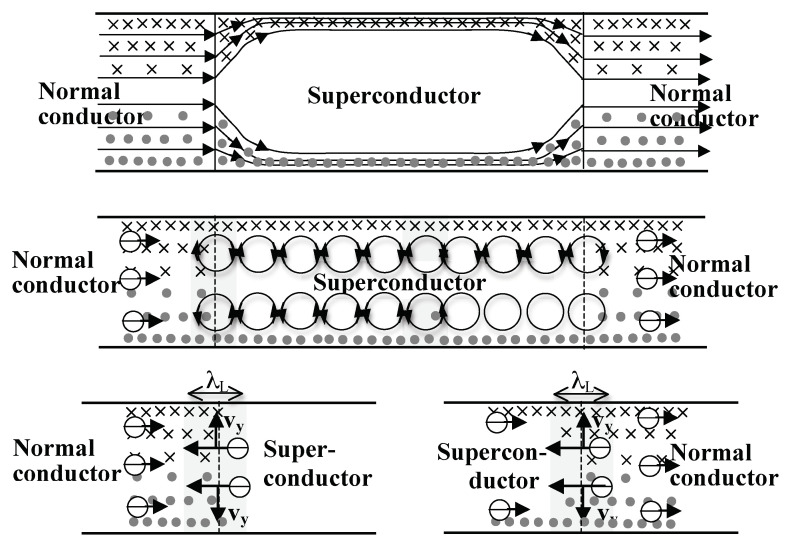
Cross-section of a superconducting wire connected to normal metal leads carrying a current from right to left (opposite to the horizontal arrows). The magnetic field generated by the current points into (out of) the paper in the upper (lower) half of the wire. The upper panel shows streamlines of electrons obtained through solution of London’s equation [29]. Note the discontinuous change in the vertical component of the velocity as electrons enter and leave the superconducting regions. This is explained through the same orbit expansion and contraction discussed earlier, as illustrated in the lower panels and explained in the text.

The same physics that explains how electrons acquire and lose the Meissner velocity in the transition of the system to and from the superconducting state explains what happens when conduction electrons enter and exit a superconducting wire connected to normal metal leads [35]. Figure 10 shows these processes in steady state. As normal electrons enter the superconducting region from the left, their orbits suddenly expand to radius $2\lambda_{L}$. This corresponds to a thrust to the left a distance $\lambda_{L}$, as shown on the left part of the lower panel of Figure 10, which through the action of the magnetic Lorentz force gives rise to a velocity ($v_{y}$) pointing upward (downward) in the upper (lower) part of the wire. This accounts for the discontinuous change in the slope of the streamlines shown in the upper panel of Figure 10 [35], which is obtained through solution of the London equations [29]. Similarly, when electrons leave the superconducting region their orbits suddenly contract, which also corresponds to a thrust to the left, as seen in the right part of the lower panel in Figure 10, and again the action of the magnetic Lorentz force explains the discontinuous slope of the streamlines shown in the upper panel of Figure 10 right side. This illustrates that the *“mechanism active in the material during the transition”* hypothesized by Cherry does not require the *“motion of a boundary”* that takes place during the phase transition but also acts when carriers enter and leave a superconducting region of fixed boundaries.

This same physics sheds light on how thermodynamic equilibrium at the phase boundary between a normal and a superconducting region in the presence of the critical magnetic field works [15], first addressed but not resolved by H. London in 1935 [36]. Electrons near the boundary must constantly transfer in and out of the superconducting region due to thermodynamic fluctuations, in the process acquiring and losing the large Meissner velocity at the phase boundary, and transferring compensating momentum to the body. This cannot possibly happen through scattering processes that would generate entropy, as would be expected within the conventional theory [15] since it is the only mechanism available within it.

Finally, we have argued that in the absence of the mechanism described here for electrons to acquire and lose the Meissner velocity, the transition between normal and superconducting states in the presence of a magnetic field would take an arbitrarily long time as the temperature approaches zero, i.e., in a magnetic field close to the zero temperature critical field [15]. To our knowledge this has not been observed. Instead, reported experimental observations [37] are consistent with the transition time being determined by Faraday’s law as discussed by Pippard [38].

## 6. Relation with BCS-London Theory

It is believed by some that London’s theory explains the Meissner effect. That is not so. The London derivation [6] leads to the equation for the current density $\vec{j}$

(16) $$\frac{\partial }{\partial t}\left(\vec{\nabla }\times \vec{j}\right)=-\frac{c}{4\pi \lambda_{L}^{2}}\frac{\partial \vec{B}}{\partial t}$$

from which the London equation

(17) $$\vec{\nabla }\times \vec{j}=-\frac{c}{4\pi \lambda_{L}^{2}}\vec{B}$$

predicting no magnetic field in the interior of the superconductor *does not follow*. Instead, the correct time integration of Equation (16) yields

(18) $$\vec{\nabla }\times \left(\vec{j}\left(\vec{r},t\right)-\vec{j}\left(\vec{r},0\right)\right)=-\frac{c}{4\pi \lambda_{L}^{2}}\left(\vec{B}\left(\vec{r},t\right)-\vec{B}\left(\vec{r},0\right)\right)$$

from which Equation (17) follows for initial conditions $\vec{B}\left(\vec{r},0\right)=\vec{j}\left(\vec{r},0\right)=0$, i.e., when a magnetic field is applied to a the system already in the superconducting state. Instead, for the Meissner effect the initial conditions are $\vec{B}\left(\vec{r},0\right)={\vec{B}}_{0},\vec{j}\left(\vec{r},0\right)=0$. So, the correct equation is

(19) $$\vec{\nabla }\times \vec{j}\left(\vec{r},t\right)=-\frac{c}{4\pi \lambda_{L}^{2}}\left(\vec{B}\left(\vec{r},t\right)-{\vec{B}}_{0}\right)$$

with solution $\vec{B}\left(\vec{r},t\right)={\vec{B}}_{0}$ and $\vec{j}\left(\vec{r},t\right)=0$ for all t, and hence the magnetic field remains in the interior of the superconductor and there is no Meissner effect.

It is also not so that BCS theory predicts the Meissner effect, as generally believed. The “derivation” of the Meissner effect within BCS theory [13] starts with the system in the BCS ground state to which a magnetic field is applied, and the resulting current that cancels the internal field is calculated through linear response theory. That is not a derivation of the Meissner effect, as we discuss in Sect. II of Ref. [26], because the system cannot be in the superconducting state with the magnetic field in the interior and then expel it. The system is initially in the normal state and only reaches the superconducting state in the process of expelling the magnetic field.

BCS theory correctly describes that the superconducting state with the magnetic field excluded has lower free energy that the normal state with the magnetic field in the interior. But the theory does not provide a mechanism for the system to go from the initial to the final state, as discussed further in the next section.

BCS theory certainly describes correctly many properties of the superconducting state, such as the pairing structure of the wavefunction, the superconducting energy gap, and macroscopic phase coherence. But that does not mean that it gives a correct description of the equilibrium state, as discussed in the next section.

## 7. Consequences for the Equilibrium State

The conventional BCS-London theory has not provided a dynamical explanation of the Meissner effect [26,39]. It is often stated by BCS practitioners that this is not necessary as long as the theory describes the equilibrium state. However, we argue that if BCS theory cannot describe the Meissner effect, its description of the equilibrium state cannot be correct either.

In particular, we argued in the previous sections that charge asymmetry is essential to understand the Meissner effect. This is also illustrated by the fact that rotating superconductors exhibit a magnetic field that is always parallel, never antiparallel, to their angular velocity [40]. The charge that is expelled and acquires the Meissner velocity through the Lorentz force is negative charge, and the backflowing normal charge has to be electrons with negative effective mass or equivalently forward-flowing holes. BCS theory does not distinguish between electrons and holes, and hence cannot describe this physics.

The understanding of the Meissner effect through the theory of hole superconductivity implies that the equilibrium state of a superconductor is different from what is predicted by BCS theory. In particular, the net result of the transition process where electrons becoming superconducting move outward and normal electrons backflow inward is a small remnant (of order 1 electron per $10^{6}$ atoms) of excess negative charge within a London penetration depth of the surface: the superfluid density in equilibrium is macroscopically inhomogeneous, as shown qualitatively in Figure 2. The quantitative description of it is given in Refs. [41,42]. Furthermore, a spin current is predicted to flow near the surface of superconductors in the absence of applied fields [30,42] in thermodynamic equilibrium. None of this physics is part of BCS theory.

## 8. Discussion

The Meissner effect is the most fundamental property of superconductors. BCS theory is universally believed to be the correct theory to describe conventional superconductivity [43]. Yet, we have argued that it does not describe the physics of the Meissner effect. The models used within BCS theory to describe superconductors do not contain the necessary physics to describe the Meissner effect because they are electron-hole symmetric and hence cannot describe the physics of charge expulsion, which requires a recognition that negative and positive charges are different. A material described by such models would not undergo a transition to a superconducting state as real superconductors do. In the presence of a magnetic field, even if electrons interacted through an effective attractive interaction, a system described by BCS theory would remain in a metastable normal state in the presence of a magnetic field, unable to expel the magnetic field to reach the phase-coherent superconducting state.

The fundamental charge asymmetry that according to the theory of hole superconductivity is at the root of superconductivity originates in the fact that electrons and protons have vastly different masses. A clear manifestation of this physics is the fact that the mean inner potential of solids is necessarily positive; in other words, electrons have to pay an energetic price to come out of a solid. The deep connection between charge asymmetry, mean inner potential, diamagnetism, and superconductivity is discussed in Ref. [44]. We have also explained the microscopic reasons for why superconductors expel negative charge [45,46].

Expulsion of magnetic field requires expulsion of charge, as recognized first by Koch 80 years ago [9,10,11] and proved mathematically in Ref. [25]. It requires expulsion of negative charge, as predicted by the theory of hole superconductivity [21]. It requires, besides outflow of charge, backflow of charge to preserve (near) charge neutrality, as first recognized by Cherry 60 years ago [12]. And it requires that superconducting electrons occupy mesoscopic orbits of radius $2\lambda_{L}$, as first recognized by Slater 85 years ago [8].

The explanation of the Meissner effect by the theory of hole superconductivity contains all those physical elements [3]. It explains the Meissner effect as a direct consequence of Alfven’s theorem [26], which states that in a perfectly conducting fluid magnetic field lines move with the fluid. The fluid that as it moves expels the magnetic field cannot carry either charge or mass, which necessitates that it is composed of electrons and holes: the electrons acquire the azimuthal speed of the supercurrent, and the holes transfer the compensating momentum to the body without scattering processes. It explains the questions posed in the Introduction: what the force that causes the development of the supercurrent in the Meissner effect is, how the growing supercurrent overcomes Faraday’s counter-emf, how the momentum of the supercurrent is compensated so that momentum conservation is not violated, how the supercurrent stops before the material develops resistance in the superconductor to normal transition, how its momentum is transferred to the body without development of Joule heat, how rotating superconductors generate magnetic fields, how electrons discontinuously change their velocity when they enter and leave a superconducting wire, etc. None of these fundamental questions is addressed by the conventional theory. We also note that the process of flow of superconducting fluid and backflow of normal fluid argued to be essential to the understanding of the Meissner effect also plays a key role in the physics of superfluid ^4^He [47].

Neither Slater nor Koch nor Cherry cited each other, indicating that they reached their physical insights independent of one another. Neither was I aware of any of their works when I developed an explanation of the Meissner effect based on the theory of hole superconductivity that contains the physical elements identified by Slater, Koch and Cherry as essential ingredients. Note in particular that I reached the conclusion that superconductors expel negative charge to the surface without relating it to the Meissner effect [21]. The coincidence of all these independent efforts is, I believe, strong evidence that these physical elements are an integral part of the correct physical understanding of the Meissner effect.

Consequences of the physics discussed here that should be experimentally detectable include that electric fields should exist around small superconducting particles in thermal equilibrium at low temperatures [48], that radial electric fields in the interior of the material should develop during the normal–superconductor transition [49] and that Alfven waves that include temperature waves as in helium’s second sound should propagate along normal–superconductor phase boundaries [50]. These have not been experimentally explored to date.

In closing, I point out that a proper understanding of the Meissner effect and of the associated predicted Spin Meissner effect [30,42] may well help with future practical applications of superconductors in ways we cannot anticipate. We also note that the theory of hole superconductivity implies that the electron–phonon interaction is irrelevant to superconductivity, contrary to what BCS theory says. This implies that efforts to raise the critical temperature by searching among compounds with light elements [51,52] are futile. Room temperature superconductors will be found when scientists focus their search for new materials on criteria consistent with physical reality [3] rather than with fiction [7].

## Data Availability

Data is contained within the article.
